# Traumatic Brain Injury and Conversion Disorder in a Veteran’s Atypical Presentation

**DOI:** 10.1192/j.eurpsy.2024.323

**Published:** 2024-08-27

**Authors:** O. Inanc, B. Garip

**Affiliations:** ^1^Gulhane Training and Research Hospital, Ankara, Türkiye; ^2^Psychiatry, Gulhane Training and Research Hospital, Ankara, Türkiye

## Abstract

**Introduction:**

Traumatic brain injury (TBI) induces cognitive and behavioral changes due to environmental impacts on brain tissue.

**Objectives:**

Highlighting the atypical TBI presentation challenging conventional diagnostics and obscured by conversion disorders.

**Methods:**

A 36-year-old male veteran, injured by a sniper rifle in 2011, presented with right ear tinnitus and monthly, unresponsive right hemicranial headaches. Seizures occurred every two weeks with no reported loss of consciousness or sensation. The gunshot wound to the neck in 2011 prompted emergency intervention, with entry and exit wounds located in the posterior lateral neck. Post-injury symptoms comprised hearing loss, tinnitus, restricted neck movement, and weakness in the right arm. Seizures persisted, accompanied by numbness and neck movement. Management included physical therapy, hyperbaric oxygen therapy (improving weakness but not tinnitus), and administration of piracetam (2400 mg/day), sertraline (100 mg/day), and ginkgo biloba (2400 mg/day). Psychiatric consultation suggested a diagnosis of “conversion disorder.”

**Results:**

Neuropsychological Evaluation: Raven Standard Progressive Matrices Test showed borderline impairment. Psychiatric Evaluation noted monotonous mimics, occasional depersonalization, reduced emotional involvement, and slowed psychomotor activity. Elevated trait anxiety was observed per the State-Trait Anxiety Inventory. Neurological Examination identified left arm weakness and impaired resting balance. Imaging Findings: F18-FDG PET/CT Scan at 1 year post-GSW showed hypermetabolism in the right frontal lobe, and at 3.5 years post-GSW demonstrated decreased glucose metabolism in the bilateral cerebellar cortex, temporal lobe, and bilateral parietal lobe.

**Conclusions:**

A high-kinetic-energy bullet passed through the right lateral base of the neck without causing apparent brain damage. Proposed is the generation of upward pressure waves in neck tissues through the transmission of kinetic energy, compressing and displacing soft tissues toward the skull. Gunshot injuries create cavities, forming high-pressure waves capable of damaging distant brain regions, leading to TBI such as crush injury, edema, and myelin and axonal damage (Courtney & Courtney, 2007). Microscopic brain damage, undetectable by current imaging, may only surface during autopsy (Yilmaz & Pekdemir, 2007). Rat studies after primary blast injuries reveal brain alterations, highlighting that high-pressure pulses can cause neuronal damage, potentially yielding related symptoms (Cernak et al., 2001). The patient’s atypical symptoms, combined with the initial conversion disorder hypothesis, underscore the need for a diagnostic paradigm shift to differentiate traumatic brain injury from other potential misnomers.

**Disclosure of Interest:**

None Declared

